# The utilization of *Blaptica dubia* cockroaches as an in vivo model to test antibiotic efficacy

**DOI:** 10.1038/s41598-021-03486-3

**Published:** 2021-12-14

**Authors:** Elliot Collins, Caleb Martin, Tyler Blomquist, Katherine Phillips, Stuart Cantlay, Nathan Fisher, Joseph Horzempa

**Affiliations:** 1 Department of Biological Sciences, West Liberty University, West Liberty, WV USA; 2 Noblis ESI, Chantilly, VA USA

**Keywords:** Bacterial infection, Drug screening, Antibiotics

## Abstract

Insects are now well recognized as biologically relevant alternative hosts for dozens of mammalian pathogens and they are routinely used in microbial pathogenesis studies. Unfortunately, these models have yet to be incorporated into the drug development pipeline. The purpose of this work was to begin to evaluate the utility of orange spotted (*Blaptica dubia*) cockroaches in early antibiotic characterization. To determine whether these model hosts could exhibit mortality when infected with bacteria that are pathogenic to humans, we subjected *B. dubia* roaches to a range of infectious doses of *Klebsiella pneumoniae*, *Escherichia coli*, *Staphylococcus aureus*, and *Acinetobacter baumannii* to identify the medial lethal dose. These results showed that lethal disease did not develop following infection of high doses of *S. aureus*, and *A. baumannii.* However, cockroaches infected with *E. coli* and *K. pneumoniae* succumbed to infection (LD50s of 5.82 × 10^6^ and 2.58 × 10^6^ respectively) suggesting that this model may have limitations based on pathogen specificity. However, because these cockroaches were susceptible to infection from *E. coli* and *K. pneumoniae*, we used these bacterial strains for subsequent antibiotic characterization studies. These studies suggested that β-lactam antibiotic persistence and dose was associated with reduction of hemolymph bacterial burden. Moreover, our data indicated that the reduction of bacterial CFU was directly due to the drug activity. Altogether, this work suggests that the orange-spotted cockroach infection model provides an alternative in vivo setting from which antibiotic efficacy can be evaluated.

## Introduction

The utilization of animal infection models is absolutely critical for the antimicrobial drug development^1^. Prior to testing in vivo, typically, compounds that either inhibit microbial growth or exhibit microbicidal activity are discovered and tested in vitro^2^. Promising antimicrobial compounds are further tested in vivo using a mammalian infection model^1,2^. Because in vitro systems cannot completely simulate host factors associated with drug stability, metabolism and excretion, bioavailability, or drug inactivation, in vivo animal infection studies are crucial during preclinical drug assessment^3,4^.

A common in vivo model used in preclinical antibiotic characterization is the neutropenic murine thigh model^5,6^. In this model, 6-week old specific pathogen-free female ICR/Swiss mice are rendered neutropenic through the use of two injections of cyclophosphamide^5^. Following the administration of anesthesia, suspensions of bacteria are injected into the mouse thigh and the mice are treated with an antibiotic. After a period of time, mice are euthanized, and mouse thighs are removed aseptically, homogenized, and this homogenate is serially diluted and plated for CFU. The pharmacokinetic and pharmacodynamic (PK/PD) data generated allow investigators to predict the potential clinical efficacy and the most effective dosing regimens of antibiotics. For instance, the amount of time the antibiotic is present in the infected animal above minimum inhibitory concentration (T > MIC) is one particular PK/PD parameter that correlates with the efficacy of beta-lactam antibiotics^7^. Analyzing PK/PD data generated by these in vivo studies allows drug developers to predict the potential efficacy in humans and eliminates the risk of conducting clinical trials using drug candidates with insufficient in vivo activity.

Insects are now well recognized as biologically relevant alternative hosts for dozens of mammalian pathogens (both bacteria and fungi) and they are routinely used in molecular pathogenesis studies^8–22^. Unfortunately, incorporation of these models into the drug development pipeline has not yet occurred in a meaningful way. We predict that in vivo PK/PD correlates for antibiotic efficacy in insect models of infection may extend to mammalian infection studies. If this were true, then utilization of insect infections early in antibiotic development may reduce the number of mammals used for experimental purposes. Therefore, the purpose of this study is to evaluate the utility of orange spotted (OS) cockroaches (*Blaptica dubia*) in early antibiotic characterization.

## Results

We have previously established that *Francisella tularensis*, *Burkholderia pseudomallei*, and *Burkholderia mallei* are lethal to tropical cockroaches at very low doses and that the tissue tropism and genetic requirements for virulence in this model are similar to that seen in mammalian models^8,15^. Based on these findings and the fact that other major multi-drug resistant (MDR) bacteria, mycobacteria, and fungi are pathogenic toward other insect species, we hypothesize that a broad range of clinically relevant human pathogens will also establish productive infections in OS cockroaches. Therefore, we sought to determine whether four model opportunistic human pathogens were capable of causing disease in OS cockroaches. To do so, we infected OS cockroaches with a range of infectious doses of *Escherichia coli*, *Staphylococcus aureus* (MRSA), *Klebsiella pneumoniae*, and *Acinetobacter baumannii* and observed whether these bacteria were able to induce insect mortality. This showed that both *E. coli* and *K. pneumoniae* were capable of establishing lethal infections in the OS cockroaches (LD50s of 5.82 × 10^6^ and 2.58 × 10^6^ respectively; Table 1 and Table S1 in the Supplemental Material). However, both *A. baumannii* and *S. aureus* bacteria were unable to produce 50% mortality in the OS cockroaches, even at infectious doses greater than 2 × 10^7^ CFU (Table 1 and Table S1 in the Supplemental Material). To ensure that these strains were at all capable of inducing lethal infection, we similarly infected *Galleria mellonella* [greater wax moth larvae, an established insect infection model^9–14,16–22^]. In the *G. mellonella* model, all bacteria were capable of mediating disease that resulted in insect mortality (LD50s of 2.42 × 10^8^, *A. baumannii*; 2.42 × 10^4^ for *E. coli*, 3.41 × 10^5^ for *K. pneumoniae*, and 9.07 × 10^5^ for *S. aureus*; Table 1 and Table S1 in the Supplemental Material). This suggests that OS cockroaches may be intrinsically resistant to particular bacterial infections. However, these cockroaches succumbed to infection from *E. coli* and *K. pneumoniae* [among others^8^] and may be useful for modeling certain mammalian infections and for preliminary antibiotic characterization studies.

**Table 1:** LD50.

Insect model	Infecting strain			
	*A. baumannii*	*E. coli*	*K. pneumoniae*	*S. aureus* MRSA
*B. dubia*	ND*	5.82 × 10^6^	2.58 × 10^6^	ND**
*G. mellonella*	2.42 × 10^8^	2.42 × 10^4^	3.41 × 10^5^	9.07 × 10^5^

*ND* not determined.

*50% lethality not produced with highest dose of 6.5 × 10^7^ bacteria per roach.

**50% lethality not produced with highest dose of 2.3 × 10^7^ bacteria per roach.

We next sought to determine if pharmacodynamic (PD) model independence extends to the OS cockroach model. Several groups have firmly established that the PD parameters that govern efficacy are largely model-independent. Differences in the physiology of bacteria grown in vitro and in vivo can lead to changes in sensitivity profiles, but there is usually very little difference in PD correlates of protection from one in vivo model to the next. This observation is fundamental to the popular neutropenic mouse thigh model wherein the host is rendered immune-incompetent so that the PD interaction between the bacterium and the drug can be analyzed in a generic in vivo environment. Within mammals, it appears that as long as the necessary PD requirements are met at the site of infection, then the treatment will be effective.

**Figure 1:**
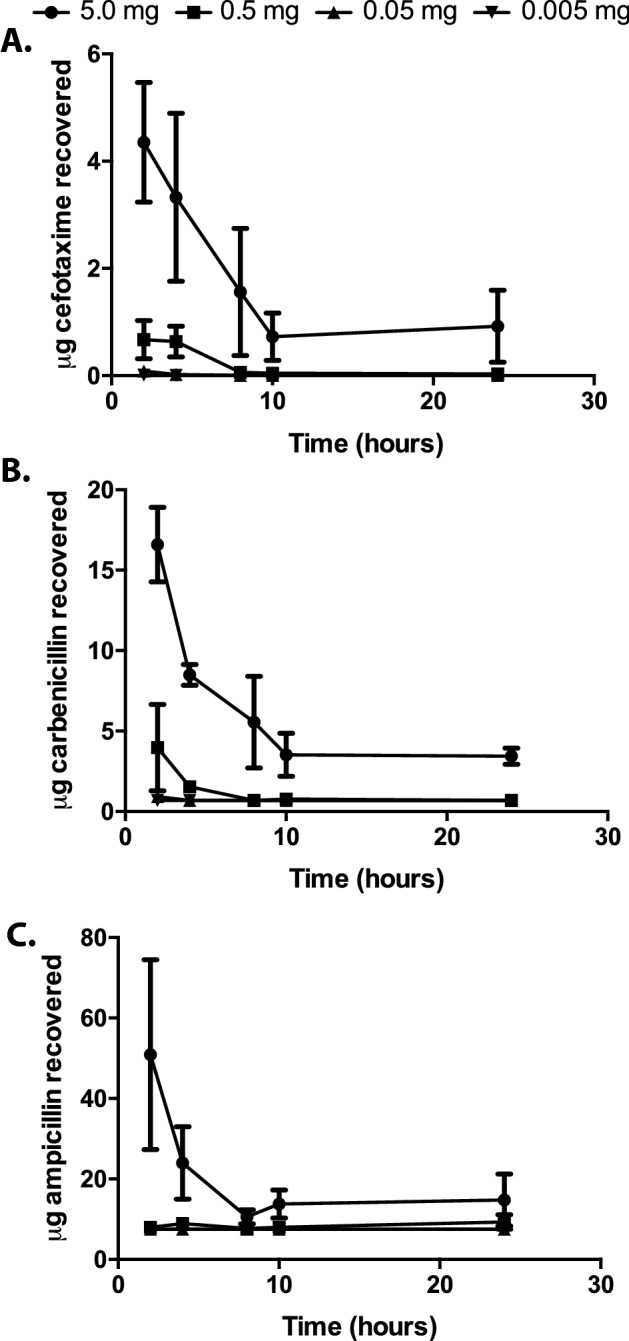
Beta lactam recovery from *B. dubia* roaches over time. Cefotaxime (**A**), carbenicillin (**B**), or ampicillin (**C**) was injected into *B. dubia* roaches at the dose indicated. At the designated time points, hemolymph was extracted and was mixed with an anticoagulant. This material was dispensed onto a disk of sterile filter paper which was placed onto TSA containing *E. coli* that had been spread-plated. Following incubation, zones of inhibition were measured; to estimate the amount of antibiotic recovered, the values for the zones of inhibition generated from the recovered hemolymph were compared to a standard curve.

One such PD metric is the drug duration (a pharmacokinetic variable). We therefore characterized the duration of three ß-lactam antibiotics in the OS cockroaches. Four different doses of cefotaxime, carbenicillin, or ampicillin were injected into OS cockroaches. At time points indicated (Fig. 1) hemolymph was extracted and spotted onto Whatman filter disks that were placed on TSA agar that had been lawn-streaked with *E. coli.* To determine the level of the ß-lactam remaining in the hemolymph, zones of inhibition were measured and compared to a standard curve. For all three antibiotics, the highest dose administered (5 mg) was still detected 24 h post-injection. For both cefotaxime and carbenicillin, the 0.5 mg dose remained above the detectable levels up to 8 h post administration. Notably, administration of any of the antibiotics used here did not affect cockroach viability, regardless of the dose (Table S2 in the supplemental material). For each of the three ß-lactam antibiotics tested, the half-life was longer in OS cockroaches than in humans or mice (Table 2). However, this difference in drug duration may not preclude the use of the OS cockroach as a model as long as this host reproduces PK/PD relationships observed in mammalian models.

**Table 2:** Half-life of cefotaxime, carbenicillin, and ampicillin for humans (as reported in the literature) and OS cockroaches.

	Cefotaxime	Carbenicillin	Ampicillin
Humans	1.1 h^43^	70 min^44^	1 h^45^
Mice	0.26 h^46^	0.3 h^47^	50 min^48^
OS cockroaches	2.3 ± 0.4 h	3.5 ± 0.1 h	2.6 h

To determine the MIC of ampicillin, carbenicillin, and cefotaxime, the broth microdilution method was used^23^. MIC values are reported in Table 3. Notably, the MIC of cefotaxime was much lower than that of ampicillin and carbenicillin for both *E. coli* and *K. pneumoniae* BLS (Table 3).

**Table 3:** Minimum inhibitory concentrations (MIC) of cefotaxime, carbenicillin, and ampicillin for *E. coli* and *K. pneumoniae* BLS.

	Cefotaxime (μg/mL)	Carbenicillin (μg/mL)	Ampicillin (μg/mL)
*E. coli*	0.0096 ± 0.0037	10 ± 0	50 ± 0
*K. pneumoniae* BLS	0.0064 ± 0.0032	250 ± 0	500 ± 0

Values shown are mean ± SD.

**Figure 2:**
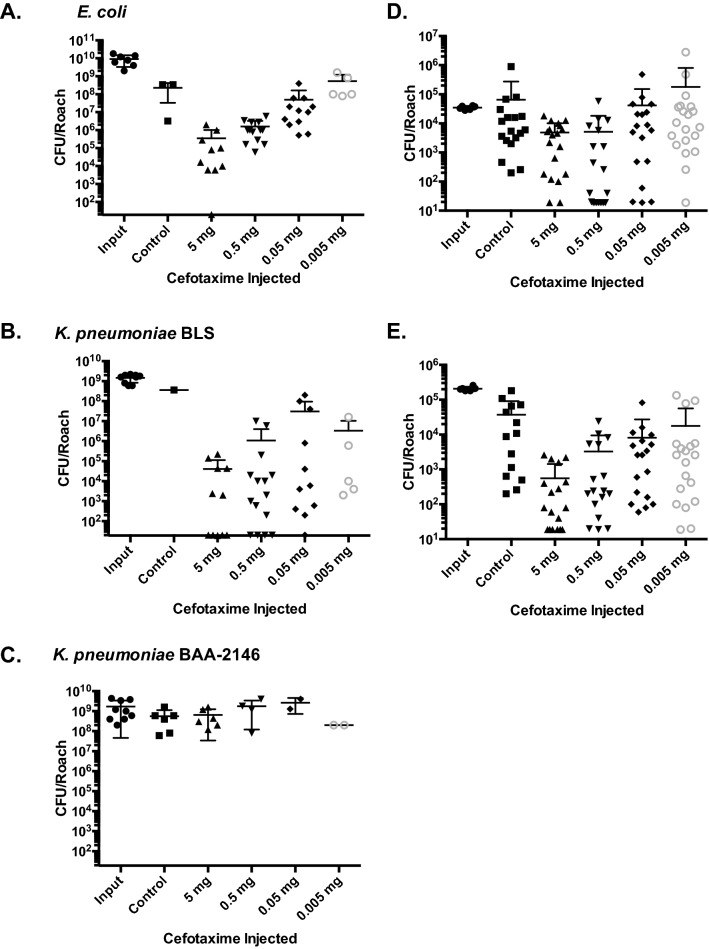
Treatment with cefotaxime reduces bacterial burden in a dose dependent manner that is specific to the activity of this drug. *B. dubia* cockroaches were infected with a lethal dose of *E. coli* (**A**), *K. pneumoniae* (BLS, Beta-lactam sensitive strain) (**B**), or *K. pneumoniae* (BAA-2146, Beta-lactam resistant) (**C**). Alternatively, *B. dubia* cockroaches were infected with a sublethal dose of *E. coli* (**D**) or *K. pneumoniae* BLS (**E**). At 24 h post infection, surviving cockroaches were euthanized and their hemolymph was isolated and mixed with an anticoagulant. This material was serially diluted and plated to enumerate CFU. CFU recovered from hemolymph was compared to input. These data were analyzed by a one way ANOVA and Tukey’s post hoc (**A**, *P* < 0.0001; *P* < 0.0001for 5 mg, 0.5 mg, 0.05 mg; *P* = 0.0784 for 0.005 mg cefotaxime. **B**, *P* < 0.0001; *P* < 0.0001for 5 mg, 0.5 mg, 0.05 mg; *P* < 0.001 for 0.005 mg cefotaxime. **C**, *P* = 0.1676. **D**, *P* < 0.0001; *P* = 0.0797 for 5 mg; *P* = 0.0007 for 0.5 mg; *P* = 0.3399 for 0.05 mg; *P* = 0.8688 for 0.005 mg cefotaxime. **E**, *P* < 0.0001; *P* < 0.0001 for 5 mg, 0.5 mg; *P* = 0.0001 for 0.05 mg; *P* < 0.005 mg cefotaxime).

Because of the low MIC for cefotaxime (Table 3) and since this antibiotic exhibited the longest duration in the OS cockroach hemolymph (Fig. 1A), cefotaxime was selected for use in the subsequent in vivo studies. We next sought to determine whether the duration of cefotaxime above the MIC correlated with a reduction in CFU in OS cockroaches that had been infected with *E. coli* or *K. pneumoniae* BLS*.* Here, we also infected OS cockroaches with a lethal dose of *K. pneumoniae* BAA 2146 (beta-lactam resistant strain) as a control as this bacterium is completely resistant to cefotaxime, and therefore should not exhibit a reduction in CFU regardless of the treatment dose. Three hours later, these roaches were injected with cefotaxime at the indicated dose (Fig. 2A–C) and were incubated at 37 °C. Twenty-four hours later, viable OS cockroaches were euthanized, and hemolymph was isolated. This material was serially diluted and plated onto solid bacterial growth medium to enumerate CFU. These data suggest that fewer *E. coli* and *K. pneumoniae* BLS bacteria were recovered from OS cockroaches from which a higher dose of cefotaxime was administered (Fig. 2A,B). However, regardless of antibiotic dose, equivalent levels of bacteria were isolated from OS cockroaches that were infected with the beta-lactam resistant *K. pneumoniae* BAA 2146 (Fig. 2C). This result indicated that the reduction in *E. coli* and *K. pneumoniae* BLS CFU was specific to the activity of cefotaxime. Moreover, for the OS cockroaches infected with either *E. coli* or *K. pneumoniae* BLS, significant insect mortality was observed in the groups that were mock-treated or treated with the lowest dose of antibiotic (Fig. 2A,B).

In a similar fashion, OS cockroaches were infected with a sublethal dose of *E. coli* or *K. pneumoniae* BLS and were treated with cefotaxime three hours later (Fig. 2D,E). Twenty-four hours later, these OS cockroaches were euthanized, and their hemolymph was extracted and serially diluted and plated on solid bacterial growth medium to enumerate CFU. These data also demonstrated a dose-dependent response of the amount of cefotaxime administered relative to the reduction of bacterial burden (Fig. 2D,E).

**Figure 3:**
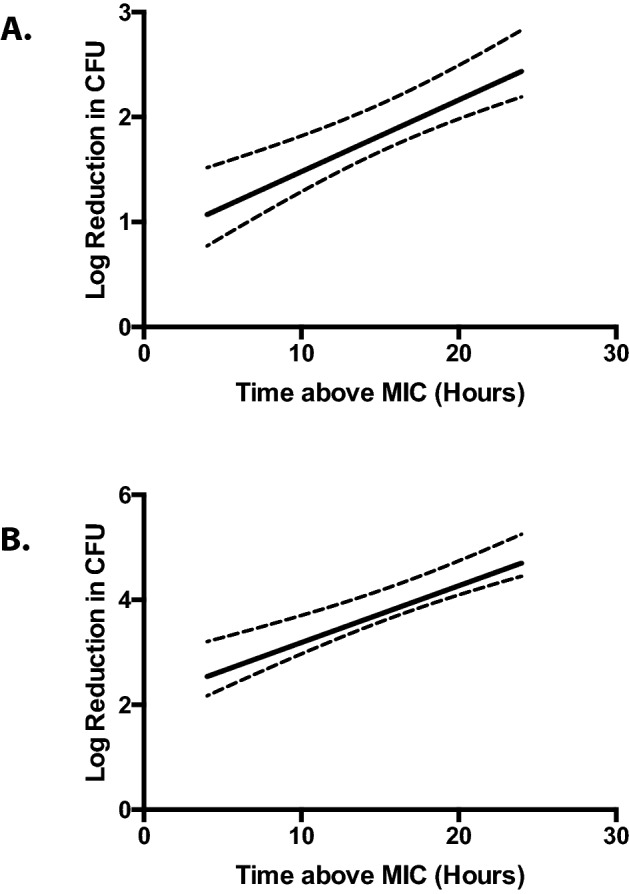
Log-reduction in CFU vs. Time > MIC exhibits a linear relationship for *E. coli* and *K. pneumoniae* BLS in the OS cockroach model. Data presented in Figs. 1 and 2 were used to extrapolate CFU reduction and Time > MIC for *E. coli* (**A**) or *K. pneumoniae* BLS (**B**). T > MIC was defined as the length of time detectible levels of antibiotic were recovered from the insects (the MIC for cefotaxime was lower than our limit of detection). The maximum value assigned for T > MIC was 24 h as this was the longest time-point from which we assayed for the presence of antibiotics from the OS cockroach hemolymph. GraphPad Prism was used to interpolate the standard curves from the mean Log-reduction in CFU values versus T > MIC for both *E. coli* (**A**) and *K. pneumoniae* BLS (**B**). The dashed lines represent the 95% confidence intervals. Adjusted R^2^ = 0.9839 (**A**) and 0.9856 (**B**).

T > MIC should exhibit a linear relationship with the log-reduction in CFU in vivo. To generate this plot for the OS cockroach model, we extrapolated the T > MIC data from the data presented in Fig. 1 and the reduction in bacterial burden from the data presented in Fig. 2. The lowest concentration of cefotaxime that we could detect by disk diffusion assay was 0.7 mg/mL, a value that was much higher than the MIC (Table 3). Because of our limitations in detecting cefotaxime from OS hemolymph, for this analysis, we defined T > MIC as the length of time we were able to recover detectable levels of antibiotic from the insects (the MIC for cefotaxime in the broth microdilution assays was lower than our limit of detection). The maximum value assigned for T > MIC was 24 h as this was the longest time-point from which we assayed for the presence of antibiotics from the OS cockroach hemolymph. This crude analysis suggests that the T > MIC of cefotaxime vs. log reduction in CFU for *E. coli* as well as *K. pneumoniae* BLS in the OS cockroach model both exhibit a linear relationship (Fig. 3A,B). Altogether, these data suggest that the OS cockroach infection model provides an in vivo setting of which β-lactam antibiotic efficacy can be evaluated on certain opportunistic pathogens.

## Discussion

Many previous studies have investigated the use of insects to model mammalian infection and to evaluate antibiotic efficacy^8–22,24–30^. Probably the most extensively utilized insect model for studies involving microbial pathogenesis and antibiotic efficacy is the waxworm, *G. mellonella*^14^*.* The waxworm model has been used to investigate traditional antibiotics, novel anti-biofilm compounds, and even bacteriophage therapy^24,25,31,32^. Although not nearly as developed as the *G. mellonella* model, the *B. dubia* OS cockroach model has potential for use as a model for pathogenesis^8^. Moreover, this current study provides further evidence that the *B. dubia* OS cockroach model has real practical potential for evaluating antibiotic efficacy in vivo. Here, we further characterized the *B. dubia* infection model by challenging this insect with various bacteria that are opportunistic human pathogens. These studies indicated that while the OS cockroaches were susceptible to infections by *E. coli* and *K. pneumoniae* bacteria, these insects exhibited minimal observable disease when infected with *S. aureus* and *A. baumannii.* These results underscore the potential limitations of this model – being unable to uniformly model infection for medically relevant human pathogens. Nevertheless, the current work showed that *E. coli* and *K. pneumoniae* were capable of causing disease in OS cockroaches, mortality was associated with a higher bacterial dose, and that bacterial burden decreased with antibiotic dose. Therefore, although limited, the OS cockroach may not only be useful for modeling a variety of infections, but this model host has potential for evaluating antibiotic efficacy in a preclinical setting prior to, or as an alternative to, mammalian models.

One of the advantages to the OS cockroach infection model is the lower price associated with these host animals. Therefore, research expenditures allocated toward infection models will provide for the utilization of a much higher sample size when using the OS cockroach host which will increase statistical power and confidence level. Facility costs are also much lower when using OS cockroaches, as these can be housed in standard laboratory settings in plastic or glass terraria. Moreover, as these animals are invertebrates, institutional oversight through an animal care and use committee is unnecessary. Finally, any ethical concerns associated with using mammals in research are eliminated by using the OS cockroaches.

Data presented here suggests that there is a linear relationship between the T > MIC of the β-lactam antibiotic, cefotaxime, vs. log-reduction in bacterial CFU of infected OS cockroaches. This is especially important since this this same metric is commonly used to predict β-lactam efficacy in vivo via the neutropenic mouse thigh model^5,6^. Therefore, adopting the OS cockroach model to screen β-lactam efficacy prior to murine studies could potentially reduce the number of mammals used for research purposes. One limitation to the current study was the lack of sensitivity of the bioassay used to estimate antibiotic duration in vivo. Utilization of HPLC–MS may have revealed lower antibiotic concentrations in the insect hemolymph far lower than our limit of detection with the bioassay. Nevertheless, the expected relationship between log-reduction in CFU and drug availability (T > MIC) was still observed here regardless of the utilization of a less sensitive approach.

## Materials and methods

### Bacterial strains and growth conditions

*Klebsiella pneumoniae* BAA-2146 (multi-drug resistant, New Delhi metallo-beta-lactamase [NDM-1] positive) *Acinetobacter baumannii* (ATCC 19606), and *Staphylococcus aureus* BAA-1556 (MRSA) were obtained from the American Type Culture Collection. The *Escherichia coli* strain used in this study was isolated from a feline urinary tract infection. *Klebsiella pneumoniae* BLS (beta lactam-sensitive) is a general laboratory strain maintained in the West Liberty University Microbiology Culture Collection^33^. Single colonies obtained from tryptic soy agar (TSA) plates were used to inoculate tryptic soy broth (TSB). These broth cultures were incubated at 37 °C until the bacteria reached stationary phase.

### Bacterial enumeration

Prior to preparing the inocula for infection studies, viable CFU/mL were determined for stationary phase cultures (data not shown). Here, broth cultures were normalized to OD_600_ = 0.3. This diluted culture was serially diluted in phosphate buffered saline (PBS) and these dilutions were plated onto TSA. After an overnight incubation at 37 °C, bacterial colonies were enumerated and CFU/mL was determined.

### Determination of MIC by broth microdilution

Minimum inhibitory concentrations were determined a modified broth microdilution^34,35^ similar to the EUCAST method (www.eucast.org). Bacteria cultured to stationary phase were used to inoculated LB broth at concentration of ~ 2 × 10^5^ CFU per well of a 96-well microtiter plate (200 μl/well). Antibiotics were serially diluted and the plates were incubated at 37 °C for ~ 16 h. A Synergy H1 microplate reader (Bio-Tek) was used to measure OD_600_. The MIC was determined to be the lowest concentration of antibiotic that inhibited bacterial growth by a difference of 0.3 OD_600_ units or more.

### Cockroach housing

*Blaptica dubia* roaches were ordered from Backwater Reptiles (www.backwaterreptiles.com) and were housed in smooth plastic terraria. Each terrarium contained a petri dish with food and hydration crystals (supplied by Backwater Reptiles) as well as several pressed paper egg cartons. Roaches were housed at 37 °C until needed for each experiment. Cockroach terraria were cleaned weekly, removing dead cockroaches and replacing food and hydration crystals. For each experiment, cockroaches were grouped (between 10 and 20) into large 150 × 25 mm petri dishes and housed at 37 °C.

### Cockroach infections

Cockroaches were infected similarly to methods previously described^8^. Bacteria were cultivated in TSB to stationary phase and were diluted in PBS to the concentration indicated. Cockroaches were injected with 10 μL of the desired bacterial suspension to the left of the midline at the base of the third tergum^8^. Cockroaches were monitored for mortality every 24 h post-infection. Mortality was determined by lack of movement following stimulation. The LD_50_ was determined by the probit analysis^36–38^.

To evaluate the effect of antibiotic concentration on the cockroach CFU burden in vivo, suspensions of bacteria were adjusted to ~ 100 × LD_50_ in PBS and the insects were subsequently infected. Three hours later, cockroaches were then injected with the antibiotic dose indicated (20 μL of each cefotaxime suspension to the right of the midline at the base of the third tergum). As a control, a group of cockroaches was infected but not treated. Cockroaches that survived at 24 h post-treatment were euthanized by decapitation, and the hemolymph was isolated^8^. To do so, the head and attached gastrointestinal tract were removed, and the hemolymph was dispensed into a microcentrifuge tube containing 20 μL 0.05% N-Phenylthiourea (anticoagulant). This material was serially diluted in PBS and then each dilution was plated to enumerate CFU^8^.

### Galleria mellonella infections

The *G. mellonella* infection model was conducted as was previously published^21,26^. *G. mellonella* larvae were purchased from Best Bet Waxworms Inc. Healthy larvae with no visible melanization were selected. Stationary phase broth cultures were diluted in PBS to the concentrations indicated. Using a 30-gauge needle, each larva was infected with 10 µL of the bacterial suspension. Infected and control larvae were incubated at 37 °C and were monitored for viability daily. The LD_50_ values were calculated in a similar fashion as was described in the *B. dubia* cockroach infection studies.

### Antibiotic rescue

In vivo antibiotic concentrations were determined using a bioassay of insect hemolymph similar to methods previously published for mammalian body fluids^39–42^. Cockroaches were injected with 20 μL of 250 mg/mL, 25 mg/mL, 2.5 mg/mL or 0.25 mg/mL Ampicillin, Carbenicillin, or Cefotaxime respectively. These concentrations were generated by serially diluting a stock antibiotic (250 mg/mL) in PBS. Intrahemoceol injections were administered to the right of the midline at the base of the third tergum^8^. Each group of cockroaches was housed in a large 150 × 25 mm petri dish at 37 °C. At the time points indicated, at least three cockroaches from each group were euthanized and hemolymph was isolated and mixed with 20 μL 0.05% N-Phenylthiourea (anticoagulant). Ten microliters of the hemolymph was dispensed onto a circular disk of sterile Whatman filter paper which was placed onto TSA containing *E. coli* (100 μL OD_600_ = 0.3 of a stationary phase culture) that had been spread-plated. TSA plates were incubated at 37 °C for 24 h. Zones of inhibition were measured in millimeters using a metric ruler. Horizontal and vertical measurements for each disk were averaged. To estimate the amount of antibiotic recovered, the values for the zones of inhibition generated from the recovered hemolymph were compared to a standard curve that was generated by dispensing known quantities of antibiotic onto the sterile Whatman circles, and carrying out the disk diffusion assays similarly as was just previously detailed here.

### Statistical analysis

GraphPad Prism was used for statistical calculations. The particular analyses used are indicated in the figure legends.

## Supplementary Information

Supplementary Table 1.

Supplementary Table 2.
